# Percutaneous cryoablation of kidney tumors after partial nephrectomy

**DOI:** 10.20452/wiitm.2024.17904

**Published:** 2024-10-25

**Authors:** Wojciech Krajewski, Maciej Guziński, Wojciech Tomczak, Łukasz Nowak, Jan Łaszkiewicz, Joanna Chorbińska, Adam Chełmoński, Katarzyna Grunwald, Bartosz Małkiewicz, Tomasz Szydełko

**Affiliations:** Department of Minimally Invasive and Robotic Urology, University Centre of Excellence in Urology, Wroclaw Medical University, Wrocław, Poland; Department of General Radiology, Interventional Radiology and Neuroradiology, Wroclaw Medical University, Wrocław, Poland; University Centre of Excellence in Urology, Wroclaw Medical University, Wrocław, Poland

**Keywords:** ablative therapy, percutaneous cryoablation, recurrence, renal cell carcinoma, renal cryoablation

## Abstract

**INTRODUCTION:**

The widespread use of ultrasound and cross‑sectional imaging has led to a steady increase in the incidental discovery of renal masses. Most of them are treated with partial nephrectomy (PN), as recommended by the European Association of Urology guidelines. However, this approach carries a risk of local recurrence. In such a case, surgical reintervention can be more challenging and is often associated with worse prognosis. In this context, percutaneous ablative therapies are a promising alternative.

**AIM:**

This study presents our experience with using percutaneous cryoablation (PCA) to manage recurrences and new masses in previously operated kidneys.

**MATERIALS AND METHODS:**

We conducted a retrospective data analysis to evaluate patients treated with PCA for tumor recurrence or residual disease in the postresection bed, excluding those with de novo or recurrent tumors in the contralateral kidney.

**RESULTS:**

A total of 23 individuals met the inclusion criteria. Of those, 14 initially underwent laparoscopic PN, and 9 were treated with open surgery. The median interval from the initial surgery to recurrence‑targeted PCA was 23 months (range, 7–228). The mean (SD) RENAL score on admission was 7.5 (1.9), and the median (interquartile range) tumor volume was 3 (1.6–4.5) ml. The median length of hospital stay was 23 hours (range, 6–55). There was no significant change in estimated glomerular filtration rate following cryoablation. All the recorded complications, except one, were grade I and resolved with hydration or treatment with nonsteroidal anti‑inflammatory drugs. No patient required dialysis in the perioperative period.

**CONCLUSIONS:**

Imaging‑guided PCA is a feasible and effective treatment option for patients with renal tumor recurrences after PN.

## INTRODUCTION 

Renal cell carcinoma (RCC) is a predominant form of kidney cancer, representing 90% of all identified renal masses and around 3% of all cancers.^1^^;^^2^ Approximately 96% of cases occur sporadically, with over half being detected incidentally.^3^ The pattern of incidental discoveries is mostly attributed to widespread utilization of ultrasound and cross‑sectional imaging studies in highly developed societies.^4^^;^^5^ This has initially resulted in a steady increase in RCC incidence over the past 20 years.^6^^;^^7^ At the moment, the curve has levelled off and a majority of newly discovered lesions are below 4 cm in size.^8^ This shift of clinical presentation along with technological advances have led to a change in the standard of care, leaving open radical nephrectomy (RN) behind.^9^ Currently, the RCC therapeutic landscape is broad and adapted according to the stage of the disease at diagnosis and the malignant potential. In general, the treatment spectrum ranges from approaches with no curative intent, such as watchful waiting, to RN. Partial nephrectomy (PN) and minimally invasive percutaneous ablative approaches reside somewhere in between.

The current European Association of Urology guidelines^10^ recommend PN as the standard method of treatment for T1 tumors.^10^ However, the reported local recurrence rate following PN ranges from 1.5% to 4.2%,^11^^;^^12^which carries a risk of additional interventions. Unfortunately, surgical reintervention in a previously operated kidney is technically challenging and associated with poor prognosis.^13^^;^^14^ Focal therapy, including percutaneous cryoablation (PCA), is a highly feasible treatment option in such cases.^15^^;^^16^

## AIM 

In this study, we aimed to share our experience with using PCA for the treatment of ipsilateral recurrences as well as newly emerged masses in previously operated kidneys.

## MATERIALS AND METHODS

###  Patients and procedure 

We conducted a retrospective data analysis of patients treated with PCA at our high‑volume University Centre. The hospital registry was searched for individuals who underwent PCA for RCC recurrence or residual disease in the post‑PN bed. Patients with de novo or recurrent tumors in the contralateral kidney were excluded. Between June 2023 and June 2024, a total of 23 cases were identified. All patients were qualified for PCA as part of shared decision‑making during multidisciplinary team meetings.

PCAs were performed by an interventional radiologist highly proficient in computed tomography (CT)‑guided interventions (MG) and an urologist with vast experience in ultrasound‑guided percutaneous renal procedures (WK). A detailed description of our approach to this procedure is provided in a separate paper.^17^ In essence, the cryoprobes were inserted percutaneously under hybrid ultrasound and intermittent CT guidance. The procedures were performed using cryoprobes manufactured by Boston Scientific (Marlborough, Massachusetts, United States) and IceCure (Cesarea, Israel). Freezing was performed with a double freeze‑thaw cycle consisting of at least 8‑minute freeze and 8‑minute thaw. 

Adverse events (AEs), defined as deviations from the expected perior postprocedural course, were systematically documented and categorized using the revised Clavien–Dindo (CD) classification for surgical complications.^18^ Follow‑up phone calls were scheduled 4 to 5 weeks after the procedure to identify any recurrences. Subsequent assessments were conducted in alignment with the planned schedule for contrast‑enhanced imaging at 3 and 6 months post‑PCA. Any AEs noted during the follow‑up were recorded and categorized in accordance with the CD classification. 

Since there is no worldwide consensus regarding timing and duration of follow‑up,^19^^;^^20^ we scheduled patients for contrast‑enhanced imaging (CT or magnetic resonance imaging [MRI]) at 3 and 6 months post‑PCA. The imaging modality was selected individually, based on the radiologist’s recommendation for optimal lesion visualization. Renal filtration was also considered, and MRI was preferred if cumulative radiation exposure was high. Subsequent follow‑up studies were scheduled based on tumor grade, previous CT scan results as well as the comorbidity burden and life expectancy.

### Data extraction and definitions

The following data were collected from medical registries: age, sex, American Society of Anesthesiologists physical status grade, body mass index, disease‑specific history, tumor characteristics, ablation procedure details, and the type of utilized equipment. Additionally, mass complexity was evaluated using the RENAL nephrometry score based on preoperative CT scans.^21^ Renal function was assessed based on estimated glomerular filtration rate (eGFR) calculated using serum creatinine (sCr) levels.

For the sake of clarity, the following definitions were used: 1) residual disease was defined as a contrast‑enhanced lesion located in the tumor bed identified during the first examination following primary treatment and corresponding with preoperative imaging findings; 2) locoregional recurrence was defined as a contrast‑enhanced lesion that appeared in the area of the tumor bed after a disease‑free period; and 3) distant recurrence was defined as a contrast‑enhanced lesion of the initially affected kidney localized outside the tumor bed.

Tumor control after PCA was defined as no signs of contrast enhancement and a lack of growth on imaging (CT or MRI). Contrast enhancement was defined as an increase in density by more than 15 Hounsfield units, or an increase in signal intensity by at least 15% on contrast‑enhanced MRI. ^22^

We consistently gathered data from both patients and physicians involved in each procedure using well‑established questionnaires. The Patient Surgical Satisfaction Questionnaire (SSQ‑8) and the NASA Task Load Index (NASA‑TLX) were used in this study. The SSQ‑8 is an 8‑item survey, validated in multiple languages, designed to measure patient satisfaction following various surgical procedures. Each item is scored on a Likert scale from 1 to 5, with low scores indicating high patient satisfaction and high scores denoting strong dissatisfaction. NASA‑TLX is a globally recognized tool used across multiple disciplines to assess the workload associated with specific tasks.^23^ It features 6 scale questions that evaluate different aspects of cognitive load, with high scores indicating higher cognitive demand. In medical settings, NASA‑TLX measures the complexity of procedures from the physician’s perspective. The cognitive demand of surgeons during PCAs was compared between previously operated and treatment‑naive individuals, with all procedures performed by the same surgical team within the same department.

Statistical analysis 

Statistical analysis was conducted using SPSS Statistics for Macintosh, version 27.0.1.0 (IBM Corp., Armonk, New York, United States). Categorical variables were described as counts and percentages, and continuous variables as meanwith SD or median with interquartile range (IQR), depending on the distribution as assessed using the Kolmogorov–Smirnov test. For time‑related measures (hours, days, months) with a non‑normal distribution, ranges were used. Significance threshold was set at a P value below 0.05. The effect of PCA on kidney function was evaluated by calculating the difference and percentage change in sCr and eGFR. Additionally, the paired samples t test was used to assess the difference between pre-and postoperative sCr levels. The Student t test was employed to analyze the results obtained from the NASA‑TLX questionnaire in order to compare procedure complexity between individuals previously treated with PN and those who were surgery‑naive.

**TABLE 1:** Demographic and clinical characteristics of the study population (n = 23)

Parameter		Value
Age, y, mean (SD)		67.1 (8.5)
Sex, n (%)	Men	7 (30.4)
	Women	16 (69.6)
BMI, kg/m2, mean (SD)		28.3 (5.2)
CCI, mean (SD)		5.4 (1.8)
ASA grade, mean (SD)		2.8 (0.4)
Antiplatelet drugs before PCA, n (%)		0
Anticoagulant drugs before PCA, n (%)		1 (4.3)
Solitary kidney, n (%)		11 (47.8)
Tumor laterality, n (%)	Right	12 (52.2)
	Left	11 (47.8)
Tumor diameter, cm, median (IQR)		2.1 (1.7–2.3)
Tumor volume, cm3, median (IQR)		3 (1.6–4.5)
RENAL score, mean (SD)		7.5 (1.9)
Type of treated mass, n (%)	Distant	6 (26.1)
	Residual	5 (21.7)
	Locoregional	12 (52.2)
Ablation modality, n (%)	IceCure	9 (39.1)
	Boston Scientific	14 (60.9)

Abbreviations: ASA, American Society of Anesthesiologists, BMI, body mass index; ParameterCCI, Charlson Comorbidity Index; IQR, interquartile range; PCA, percutaneous cryoablation

**TABLE 2:** Summary of adverse events recorded during and after the procedure

Complication	Patients, n	Management	Clavien–Dindo grade
General malaise	1	Observation	I
Pain of moderate intensity	4	NSAIDs	I
Episode of mild hypotension	1	Lower dose of antihypertensive drugs	I
Hematuria without clots	2	Hydration	I
Hematuria with clots	2	Hydration	I
UTI	1	Empiric antibiotic	II

Abbreviations: NSAIDs, nonsteroidal anti‑inflammatory drugs; UTI, urinary tract infection

### Ethics 

This study protocol was approved by the local institutional review board (KB610/2024), and all patients provided written informed consent to participate. This study complies with the ethical guidelines outlined in the Declaration of Helsinki, originally established in 1975 and revised in 2000.

## RESULTS 

A total of 23 individuals met the inclusion criteria. Of those, 14 initially underwent laparoscopic PN, while the remaining 9 were first treated with open PN. Eleven patients presented with a solitary kidney, either anatomically or functionally. In 5 cases, the contralateral kidney had been previously removed due to malignancy (4 cases of RCC and 1 case of upper tract urothelial cancer). At the time of qualification for primary kidney tumor surgery, the recorded pathological stages were pT1 in 16 cases (69.6%), pT2 in 6 cases (26.1%), and pT3 in 1 case (4.3%). The median time from the initial surgery to recurrence‑targeted PCA was 23 months (range, 7–228). The mean (SD) RENAL score on admission was 7.5 (1.9), and the median (IQR) tumor volume was 3 (1.6–4.5) ml. Baseline characteristics of the included patients are provided in TABLE 1.

The median duration of hospital stay was 23 hours (range, 6–55). Thirteen patients were discharged on postoperative day 0, and 10 were discharged on postoperative day 1. In 5 cases, hospitalization extended overnight due to a severe comorbidity burden. The other 5 patients stayed in the hospital in compliance with an older protocol rather than due to medical necessity.

Among the study participants, the following pre‑PCA biopsy results were recorded: presence of malignant tissue was confirmed in 12 patients (52.2%), of whom 8 had clear cell carcinoma, 3 had papillary RCC, and 1 had chromophobe RCC. In 6 individuals (26.1%) biopsy was inconclusive; however, the radiological imaging findings were unequivocal in all these patients, and a majority of them presented with locoregional recurrences that were previously confirmed to be malignant. In 5 cases (21.7%) biopsy was not performed because of evident residual disease.

With respect to anesthesiology, 14 PCAs were performed using remifentanil sedation, while 9 procedures were conducted under local anesthesia only. The choice of the anesthesia regimen was determined by the protocol at the time. Of the patients who underwent the procedure under local anesthesia, none required additional sedation or analgesics.

The recorded AEs are listed in  TABLE 2. Notably, no patient experienced clinically significant perirenal hematomas, urinomas, bowel injuries, or other severe complications, either during or after the procedure.

**FIGURE 1:**
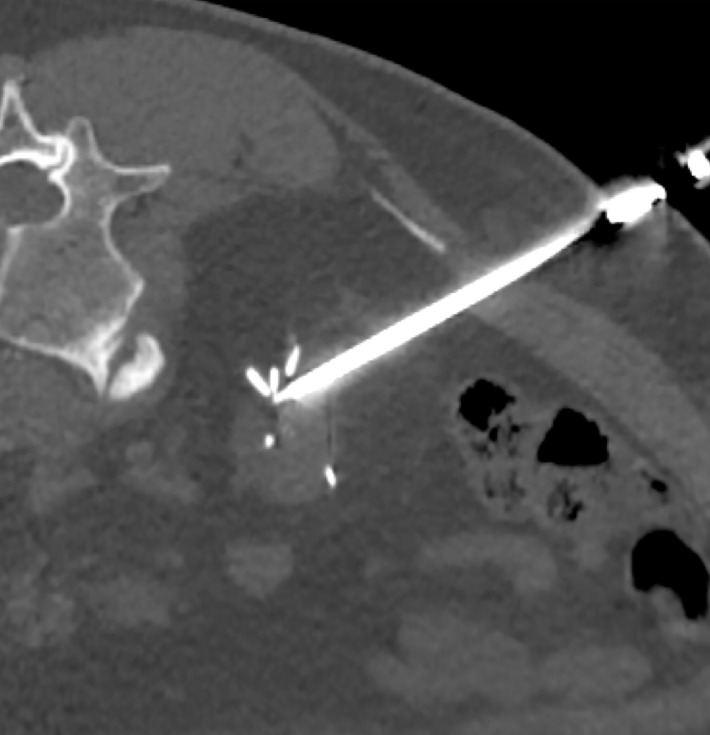
Cryoprobe positioned in a tumor bed filled with staples

In 13 patients, postoperative results were confirmed as complete based on immediate CT scans. However, in 4 cases, technical issues prevented the iceball from covering the thin edge of the viable tumor. In 1 patient, numerous metal staples in the tumor bed hindered optimal positioning of the cryoprobes FIGURE 1. In another patient, the bowel was closely overlapping the tumor and was not compliant to hydrodissection, probably due to postoperative adhesions after the primary surgery  TABLE 2. In the other cases, completeness of the procedure was very likely, but due to altered anatomy, it was impossible to confirm this fact authoritatively on immediate CT.

**FIGURE 2:**
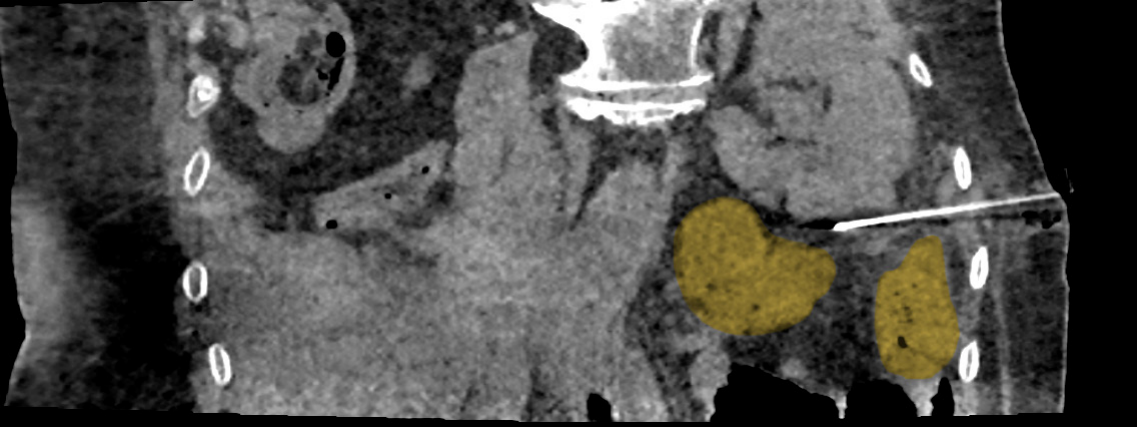
Intestine in close proximity to the tumor (marked in yellow)

As of July 22, 2024, 3‑month follow‑up CT scans were available for 18 patients. Of those, 11 showed no signs of viable tumor. The abovementioned 4 patients with nonradical ablation displayed small areas of contrast‑enhanced tissue. After extensive counselling, all of those patients opted for active surveillance. The other 3 individuals presented small ambiguous contrast uptake foci at the PCA site. Notably, all of them were treated for locoregional recurrence. It was decided to implement close radiologic surveillance in these individuals. During the follow‑up, 2 patients died of unrelated reasons, and no cases of metastatic spread were observed.

The mean (SD) preprocedural eGFR was 78 (23) ml/min/1.73 m2 (range, 44–123). At the 3‑month follow‑up, the mean (SD) eGFR was 76 (22) ml/min/1.73 m^2^ (range, 41–119), showing no significant difference as compared with the baseline value. This was also the case in the patients with a solitary kidney and those with chronic kidney disease (CKD). However, at the 3‑month follow‑up, 1 patient experienced a decrease in eGFR of more than 20 ml/min/1.73 m^2^.Additionally, 2 patients with stage II CKD progressed to stage III.

The level of patient satisfaction measured by the SSQ‑8 questionnaire was high, with a mean (SD) value of 1.34 (1.19). All patients explicitly indicated that they would recommend this treatment methods to others. The mean (SD) NASA‑TLX score was 11.3 (5.33), reflecting a significant increase in the complexity of post‑PN PCA, as compared with procedures conducted on surgery‑naive kidneys. Detailed scores for each NASA‑TLX domain are presented in TABLE 3.

## DISCUSSION 

When treating renal cancer with curative intent, kidney‑sparing modalities should be the first‑choice approach. This is particularly important for individuals presenting with a solitary kidney, either anatomically or functionally. However, as compared with RN, these procedures car‑ ry a higher risk of incomplete mass removal and tumor recurrence, both at the primary tumor bed and in other locations within the kidney.^24^^;^^25^ In addition to the need for satisfactory cancer control, it is vital to preserve sufficient renal function to avoid end‑stage renal disease (ESRD), which is associated with increased rates of cardiovascular events, hospitalization, and death.^26^^;^^27^ Moreover, in patients with ESRD, the risk of developing RCC in longer follow‑up increases 10‑fold, potentially leading to a vicious cycle. ^28^

Secondary kidney surgeries are remarkably more challenging than initial ones. Altered anatomical relations, scars, adhesions, and, last but not least, suturing and stapling material may render effective surgery impossible. Moreover, all these factors substantially increase the risk of postoperative complications. In such cases, patients may benefit from percutaneous focal approaches, including microwave, radiofrequency, and cryoablations. From a theoretical stand‑ point, these procedures allow for precise introduction of ablation probes, with a relatively low risk of AEs. Also, incomplete ablation can be easily repeated without major risks.

We presented a case series in which PCA was used to treat kidneys that initially had been operated on classically. The analyzed procedures turned out to be more difficult than PCAs performed in patients with no previous history of PN. The NASA‑TLX scores were generally high, but they were significantly higher for post‑PN procedures than for PCAs conducted on surgery‑naive kidneys. Particularly, the scores associated with physical demand, effort, and frustration were significantly elevated. Subjectively, the primary reasons were altered kidney anatomy and restricted tumor visualization on both CT and ultrasound. As a general rule, adhesions restrict both respiratory and passive movements of the kidneys, facilitating precise needle insertion. On the other hand, adhesions, scar tissues, and suturing material are much more difficult to penetrate. Also, in some individuals, surrounding tissues are much more difficult to displace with hydrodissection. Finally, designing the iceball and whole tumor coverage is considerably more intricate in patients with a history of prior surgery.

**TABLE 3:** NASA Task Load Index scores for procedures performed in the study population, as compared with procedures conducted in surgery‑naive patients

Domain	Post‑PN	Surgery‑naive	*P *value **a**
	(n = 23)	(n = 62)	
Mental demand	10.8 (5.22)	9.6 (4.14)	0.48
Physical demand	10.8 (5.47)	7.3 (3.15)	0.05
Temporal demand	10.6 (5.42)	10.2 (4.18)	0.73
Performance	6.5 (4.4)	5.9 (2.76)	0.99
Effort	14 (4.29)	10.5 (4.37)	0.06
Frustration	15.3 (3.8)	9.9 (4.82)	0.03

Data are presented as mean (SD).

a Values were compared using the Student t test. Abbreviations: PN, partial nephrectomy

The effectiveness of renal tumor treatment should be assessed on the basis of risk group allocation with a corresponding follow‑up time‑table. However, the available recommendations only apply to primary tumors. Tailoring postablative follow‑up is more challenging since biopsy results are not always available. Even when they are, the biopsy diagnostic yield is far less conclusive than the pathology report of a traditionally resected tumor. Ultimately, radiologic assessment is less reliable and way more complex than in therapy‑naive kidneys. As previously reported, early post‑PCA lesions in the region of interest might display signs of contrast enhancement not indicative of viable malignant tissue (eg, rim enhancement or discrete tumor enhancement).^29^^;^^30^^;^^31^ There is no established consensus regarding particular follow‑up strategy. In general, the trend is to perform 3 to 5 imaging studies during the first year, and then at increasing intervals up to 5 years. In our center, we implemented a 3‑ and 6‑month follow‑up schedule for every patient, and further examinations were arranged individually. In patients with an ambiguous, nonspecific contrast uptake at the PCA site, closer radiologic follow‑up should be implemented.^32^ However, in the cases with evident nonradical ablation, a salvage surgery may be technically demanding, and the risk of serious complications is high.^33^ Therefore, after proper counselling, such patients might be candidates for re‑PCA or for surveillance protocols.

Abdelsalam et al^34^ published the results of the largest‑to‑date cohort treated with thermal ablation following PN. The reported ablation technical success rate was 100%, although only 14 out of 74 procedures were PCAs. Additionally, tumor recurrence in the surgical bed was an exclusion criterion. Nevertheless, in a long follow‑up, this study displayed low recurrence and complication rates following ablative therapy. Specifically, local recurrence was detected in only 4.6% of lesions over a median follow‑up of 60 months. Additionally, 7 patients (9.5%) experienced procedure‑related AEs. Of these, 2 were managed conservatively, 2 developed urinary tract infections (UTIs) treated with antibiotics, and 3 required ureteral stent or catheter placement. As PCA procedures focused on the tumor bed are more intricate than those performed for distant recurrences, higher AE rates can be expected. This was also the case in our study—we noted relatively more AEs than in the aforementioned cohort. However, all the recorded complications, except one, were classified as grade I and resolved after hydration or administration of nonsteroidal anti‑inflammatory drugs. The only grade II complication was a UTI treated successfully with empiric antimicrobial regimen. No complications higher than grade II occurred, meaning no surgical or endoscopic interventions were conducted. None of the patients required prolonged hospitalization due to perioperative issues. Brassier et al^35^ further investigated procedure‑related AEs by comparing surgical and oncological outcomes between patients with recurrent RCC treated traditionally or with thermal ablation. They found that the probability of experiencing any post‑treatment AEs was lower after PCA than after PN (odds ratio, 0.07; 95% CI, 0.01–0.34; P = 0.001).

Various methods can be applied to analyze the impact of treatment on renal function, including dynamic renal scintigraphy, estimation of renal parenchyma volume (RPV) loss, and monitoring changes in the sCr level or eGFR. Woldu et al^36^ compared nephron‑sparing procedures by evaluating the associated loss of RPV. Using rendering software, they calculated the preoperative and postoperative RPV of the tumor‑bearing kidney, excluding the tumor in preoperative images and the postsurgical or ablative defect in postoperative images. They showed that RPV loss was significantly higher for PN than for PCA. The former showed a mean RPV loss of 16.5%, whereas for PCA, it was only 8.1%. In our study, we used changes in sCr level and eGFR as the end points. Values at 2 different time points were used. The most recent sCr measurement, taken within 3 months after the procedure, was used to evaluate the early‑to‑intermediate influence of the procedure on kidney function. No significant differences were identified in whole population. However, 2 patients with CKD stage II progressed to stage III. Similar findings have been reported in other studies, indicating that individuals with a baseline low eGFR are at a higher risk of disease progression.^37^ Nevertheless, the risk of progression still appears to be lower than that associated with classic surgical management.^35^^;^^38^

### Limitations 

Several limitations of the study need to be acknowledged. First, the study population was small and heterogenous, and a classic survival analysis was not performed due to a short follow‑up. Additionally, as the follow‑up period extends, the likelihood of recurrence is expected to increase. Secondly, in some patients, tumor biopsy results were inconclusive or unavailable, which additionally hinders the evaluation of the reported outcomes.

## CONCLUSIONS 

Imaging‑guided PCA is a feasible and effective treatment option for patients with renal tumor recurrences after PN. Its effectiveness and low risk of complications make it an alternative to subsequent surgical procedures, particularly for individuals with significant comorbidities or those with a single kidney.
